# Prenatal regression of the trophotaenial placenta in a viviparous fish, Xenotoca eiseni

**DOI:** 10.1038/srep07855

**Published:** 2015-01-19

**Authors:** Atsuo Iida, Toshiyuki Nishimaki, Atsuko Sehara-Fujisawa

**Affiliations:** 1 Department of Growth Regulation, Institute for Frontier Medical Sciences, Kyoto University, Kawahara-cho 53, Shogo-in, Kyoto 606-8507, Japan; 2 Department of Anatomy, Kitasato University School of Medicine 1-15-1 Kitasato, Sagamihara Kanagawa 228-8555, Japan

## Abstract

The trophotaenial placenta is a branching, ribbon-like structure that extends from the perianal region of the embryo in viviparous teleost fishes belonging to the family Goodeidae. It is a hindgut-derived pseudoplacenta, which contributes to absorbing maternal nutrients during the prenatal stage. The trophotaeniae are known to reduce at birth; however, no previous study has evaluated the removal mechanisms. We report here the analysis of the trophotaeniae using the goodeid fish species *Xenotoca eiseni*. The *X. eiseni* trophotaenia consists of an epidermal cell layer, mesenchyme, vasculature, and circulating erythrocytes. The trophotaeniae had preliminary regressed when the embryo was born. Immunohistochemistry indicated that caspase3-activated cells with fragmented nuclei are present in the regressed processes of the fry immediately after birth, but not in the vasculature and blood cells. This finding suggests that the trophotaenia is rapidly resorbed by apoptosis in the last phase of the pregnancy and that its circulatory pathway is maintained. Such prenatal regression of pseudoplacentae has not been reported in other viviparous vertebrates. On the other hand, similar apoptotic remodeling in the gut has been reported in amphibians, which is regulated by thyroid hormone. Thus, apoptotic regression of the trophotaeniae might occur in a manner similar to amphibian metamorphosis.

Viviparous animals are widely distributed in the extant vertebrates^1^. In mammals, all species, excluding the monotremes, show embryonic growth and development within the female body, supported by the provision of maternally derived nutrients. For nutrient absorption, mammals have a placenta and umbilical cords fused to the mother's body. In the case of non-mammalian vertebrates, the viviparous reptile *Trachylepis ivensi* has a presumed homolog to the mammalian placenta and umbilical cord^2^. In addition, some viviparous cartilaginous fishes possess a yolk sac-derived pseudoplacenta that forms during embryonic development in the mother's body^3^. These diverse viviparous reproduction systems are considered to have evolved independently in vertebrates.

Over 500 species of teleost fish have been identified as viviparous, and in some species, the embryo weight increases during pregnancy^4^. Thus, these fishes likely possess the specific machinery required to absorb maternally derived nutrients. In particular, the order Cyprinodontiformes includes approximately 170 viviparous species^5^,^6^,^7^,^8^. In this study, we focused on a viviparous teleost species that belongs to the family Goodeidae (*Xenotoca eiseni*). *X. eiseni* is distributed in the lakes and rivers of the Central Plateau of Mexico and is known to have a unique structure, the “trophotaenial placenta,” which is a pseudoplacenta that functions to absorb the maternally derived nutrients^9^. Their eggs hatch in the ovary of the mother's body, where the embryos develop until birth. This absorption of nutrients via the trophotaeniae allows for the *X. eiseni* offspring to be born at a more advanced stage relative to that observed in oviparous and ovoviviparous fishes. A previous study showed that all viviparous species belonging to the family Goodeidae, excluding *Ataeniobius toweri*, possess a trophotaenial placenta during their development^10^,^11^. These trophotaeniae were observed as a hindgut-derived ribbon-like structure that extends from the perianal region of the embryo. Given that the trophotaenia is not fused to the mother's body, these goodeid embryos likely receive the maternally derived nutrients provided from the ovarian lumen in a secreted form^12^,^13^. Therefore, this structure is not a homologous organ of the mammalian placenta and umbilical cord, neither anatomically nor functionally.

The trophotaenial placenta is only transiently required for absorption of the maternally derived nutrients during development. Since the offspring can ingest food orally soon after birth, the trophotaeniae become unnecessary. In fact, the trophotaeniae of goodeid embryos show preliminary regression at birth and then disappear within a few days^5^,^14^. Thus, the removal machinery for the trophotaeniae begins to function at the prenatal stage. However, no previous studies have elucidated the specific regression mechanisms of the trophotaeniae. Therefore, we used *X. eiseni* as a model viviparous goodeid species to analyze the trophotaenial placenta in detail^14^,^15^.

## Results

### Observation of the trophotaenial placenta in the developing embryo of *X. eiseni*

**Figure 1:**
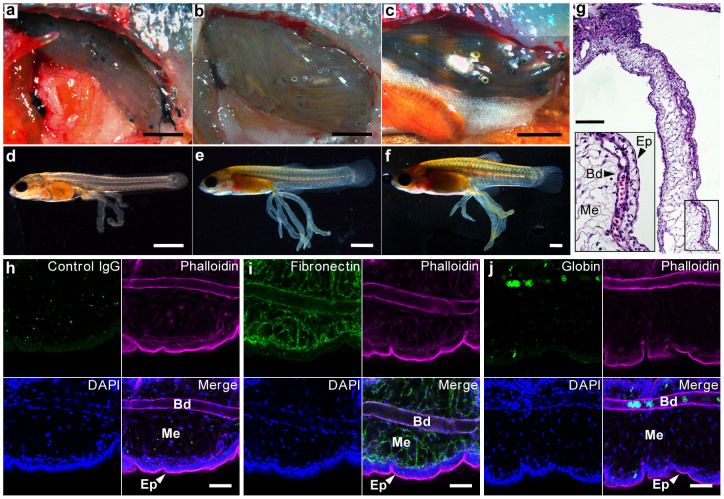
Histological analysis of the trophotaenial placenta. (a–c). The ovaries of pregnant females at the 2nd (a), 3rd (b), and 4th week (c) post-mating. Scale bar: 5 mm. (d–f). The embryo extracted from the ovaries at the 2nd (d), 3rd (e), and 4th week (f). Scale bar: 1 mm. (g). Hematoxylin-eosin-stained section of the trophotaeniae of a 4th-week embryo. Scale bar: 100 μm. (h–j). Fluorescent immunochemistry to visualize the structure of the trophotaeniae in the 4th-week embryo. Scale bar: 50 μm. Bd, blood vessel; Ep, epidermal cell layer; Me, mesenchyme.

We purchased *X. eiseni* from a commercial supplier, and the fish were bred in our fish facility. The pregnancy duration of *X. eiseni* was approximately five weeks (34–39 days) under our breeding conditions. The embryos were obtained from pregnant *X. eiseni* females at the 2nd, 3rd, and 4th weeks after mating (Fig. 1a–1c). They showed different degrees of trophotaeniae elongation from the perianal region, depending on the stage of embryonic development (Fig. 1d–1f). The trophotaeniae were not fused to the maternal tissues, and no decidual-like structure was observed on the ovarian lumen.

Histological analyses of sections of the 4th-week embryos stained using hematoxylin-eosin (HE) indicated that the trophotaeniae have a complicated structure, consisting of an epidermal cell layer, mesenchyme, vasculature, and blood cells (Fig. 1g). The processes were continuous with the gastrointestinal submucosa or epidermis of the fry (Supplementary Fig. 1), similar to a previous description of this fish family^5^. However, the precise origin of each component in the trophotaeniae could not be identified in this study. Fluorescent microscopy revealed that the epidermal cell layer could be labeled using a fluorescent-conjugated phalloidin marker, which was found to be associated to filamentous actins. The fibronectin-rich mesenchyme was surrounded by the epithelial-like component. Globin-positive erythrocytes were detected in the lumen of the phalloidin-associated vasculature walls (Fig. 1h–1j). Live observations indicated that the blood cells and the plasma component were circulating in the processes throughout the embryonic body (Supplementary Movie 1). These observations indicated that the *X. eiseni* embryo possesses a typical trophotaenial placenta that is similar to that of other viviparous species belonging to the family Goodeidae^12^,^13^.

### Prenatal regression of the trophotaenial placenta in the fry

**Figure 2:**
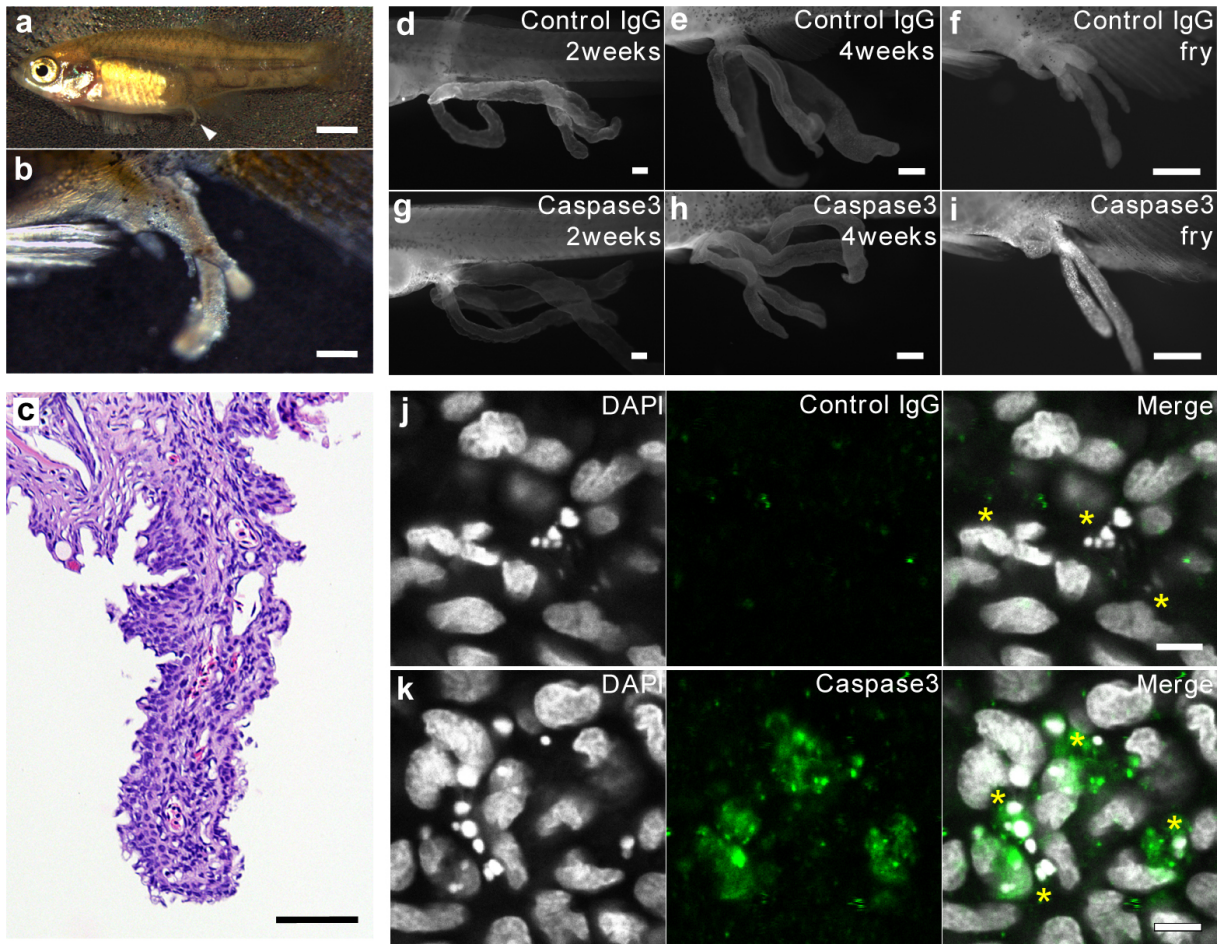
Programmed cell death in the regressed processes. (a–b). Full image of a fry immediately after birth (a) and an enlarged image of the regressed process, a vestige of the trophotaenia in the fry (b). Scale bar: 2 mm (a) and 200 μm (b). (c). Hematoxylin-eosin-stained section of the regressed processes in the fry. Scale bar: 50 μm. (d–i). Fluorescent immunochemistry to detect the apoptotic cells in the trophotaenial placenta (2nd and 4th weeks) and the regressed processes (fry). Scale bar: 500 μm. (j–k). Confocal microscopy of fluorescent immunochemistry to detect the apoptotic cells in the regressed processes. The asterisks indicate the apoptotic cells defined by fragmented nuclei. Scale bar: 5 μm.

**Figure 3:**
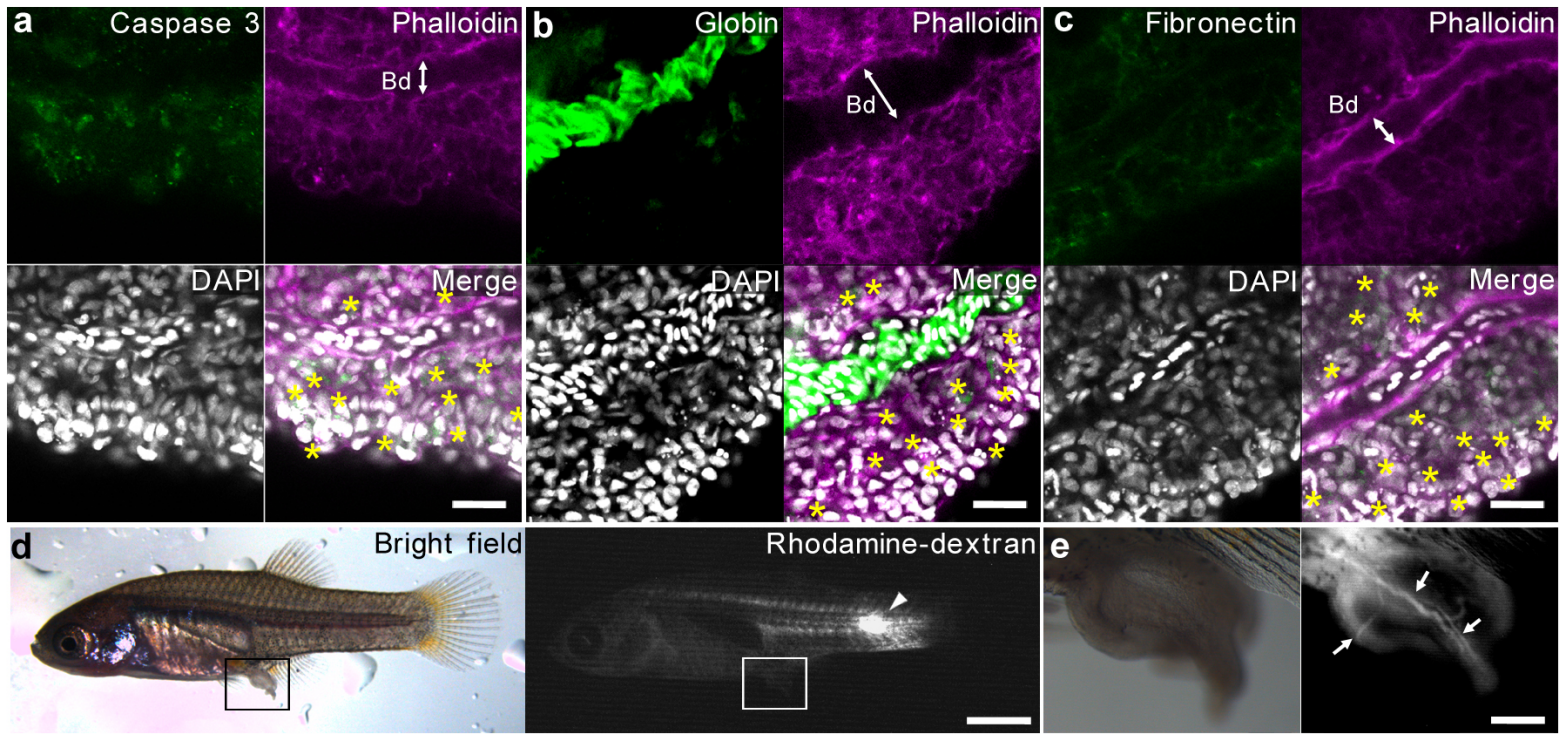
Maintenance of the circulatory pathway during the regression. (a–c). Confocal microscopy of the fluorescent immunochemistry to visualize the structure of the regressed processes. The asterisks indicate the apoptotic cells defined by fragmented nuclei, which are merged with the active-caspase3 in (a). Scale bar: 20 μm. (d). Micro-angiography of the vascular network of the regressed processes. The arrowheads indicate the dextran-injected site. Scale bar: 2 mm. (e). Enlarged image of the regressed processes. The arrows indicate the intravascular lumen visualized by dextran. Scale bar: 200 μm. Bd, blood vessel.

We observed shrunken processes at the perianal region of the fry immediately after birth, which were considered to be residues of the trophotaeniae (Fig. 2a & 2b). Histological observations showed that the epidermal cell layer and mesenchymal structures were lost in the regressed processes. The vasculature showed a snaking pattern, and blood cells were observed in the vascular lumen (Fig. 2c & Fig. 3b). The developmental stage of the fry was estimated to be at around the 5th week post-fertilization. The 4th-week embryos still had complete processes with no signs of regression (Fig. 1f & 1g). These observations suggested that the trophotaeniae undergo rapid regression in the mother's body at the last phase of pregnancy. Because of the important role that the trophotaenial placenta plays for uptake of maternally derived nutrients during embryonic development in the mother's body, prenatal regression should be controlled under a firm regulation mechanism.

### Activation of the apoptosis pathway in the regression process

**Figure 4:**
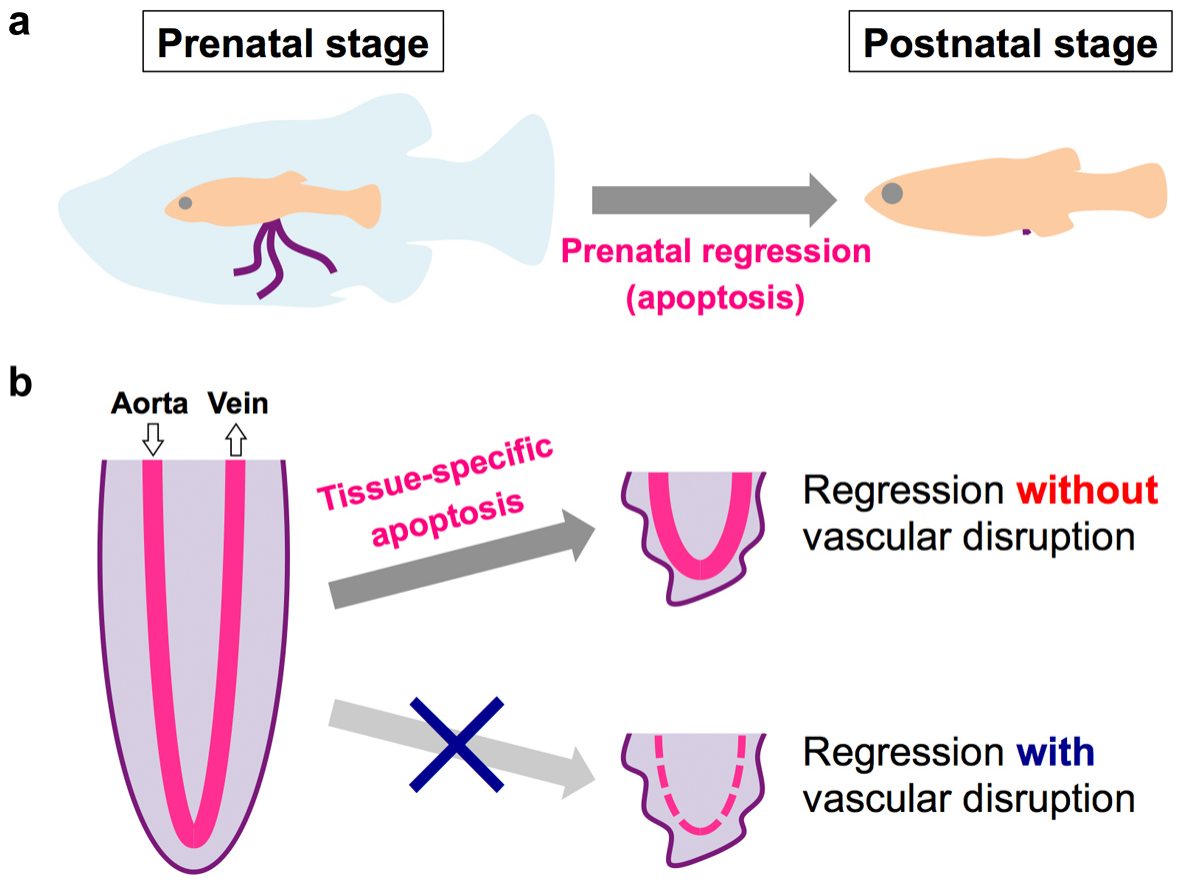
Prenatal regression in the trophotaenial placenta of *Xenotoca eiseni*. (a). Schematic illustration summarizing the model of trophotaenial placenta changes during development. The trophotaeniae (purple) begin to disappear at the last stage of pregnancy before birth. This “prenatal regression” process is regulated by apoptosis. (b). Schematic illustration summarizing the tissue-specific regression. The trophotaenia prenatally regressed without disruption of the circulatory pathway (vasculature in the trophotaenia, magenta).

Apoptosis, a process of programmed cell death involving nucleus fragmentation, widely contributes to disease development, physiological tissue turnover, and morphogenesis during development^16^,^17^. Caspase3 activation is a major marker of apoptosis-related cell death^18^. To determine the role of apoptosis in the prenatal regression of the trophotaeniae, the regressed processes were stained using anti-activated caspase3 antibody and 4′6-diamidino-2-phenylindole (DAPI). Fluorescent microscopy showed positive active-caspase3 antibody labeling, indicating the presence of some apoptotic cells in the regressed processes of the fry (Fig. 2f & 2i). These cells were not detected in the elongated trophotaeniae of the 2nd- or 4th-week embryos (Fig. 2d, 2e, 2g, & 2h). In addition, confocal microscopy revealed that the active-caspase3 signals were merged with fragmented nuclei in the regressed processes (Fig. 2j & 2k). These observations indicated that the trophotaenial placenta of *X. eiseni* prenatally regresses by a process of apoptotic cell death (Fig. 4a).

### Tissue-specific apoptosis activation during prenatal regression

Confocal microscopic observations indicated the presence of apoptotic cells, as determined by active-caspase3 staining and fragmented nuclei, in specific tissues of the regressed processes: the epidermal cell layer and mesenchyme (Fig. 3a–3c). In addition, the fibronectin-associated mesenchyme showed irregular shapes and there was no phalloidin-labeled epidermal cell layer in the regressed processes (compare Fig. 3c to Fig. 1i). These results suggested that the epidermal and mesenchymal cells might receive the apoptosis signal. In contrast, the vasculature and blood cells appear to have low sensitivity or to be resistant to these signals of apoptotic cell death. These results suggested that the blood circulation system of the trophotaeniae survives and maintains its function during the regression process. To verify the function of the circulatory system in the postnatal stage, fluorescent-conjugated dextran was injected into the cardinal vein of the fry. The fluorescence signal demonstrated a vascular network not only in the body but also in the regressed processes (Fig. 3d & 3e). Micro-angiography indicated that a closed vascular network from the body to the trophotaeniae was maintained during the regression process. In addition, live observations indicated that the intravascular blood cells circulated through the regressed processes in a manner similar to that observed during the prenatal stage (Supplementary Movie 2 & 3). These results confirmed that the circulatory pathway in the trophotaenial placenta is indeed maintained during the regression phase (Fig. 4b).

## Discussion

In this study, we elucidated the apoptotic regression process for the postnatal removal of the trophotaenial placenta using the goodeid fish species *X. eiseni*. To the best of our knowledge, there are no previous reports about such apoptotic regression in placentae or pseudoplacentae that function to absorb maternally derived nutrients during the development of viviparous vertebrates. In humans, a few apoptotic cells are also detectable in the placenta throughout pregnancy^19^. However, there are no reports of any positive roles of apoptosis in the fetal development of mammals, and an increase in apoptotic cells instead indicates a pathological process that could result in placental disorders^20^. In the case of amphibians, an apoptosis-regulated mechanism of tissue/organ remodeling occurs in the tail, brain, and intestine during metamorphosis^21^. As described previously, the trophotaenial placenta of *X. eiseni* is derived from the hindgut. Thus, the apoptotic regression and morphological changes occurring in the goodeid embryo might represent a similar mechanism to that of amphibian metamorphosis.

We did not identify the specific upstream pathway responsible for apoptosis activation. Previous studies reported that apoptosis is induced by an intrinsic and/or extrinsic pathway^22^. One possibility is that an extrinsic signal is transmitted from the mother to the embryo. In this scenario, a direct trigger for apoptosis activation is provided in the form of maternal elements. Alternatively, the embryo might autonomously regulate the apoptosis by an intrinsic and/or extrinsic pathway. One such candidate is the thyroid hormone (TH), which contributes to amphibian metamorphosis and induces apoptosis in the larval intestine^21^. TH and its receptors are conserved in teleosts and are expressed in the embryonic stage and contribute to developmental changes in the gut and other organs^23^,^24^. Thus, these molecules are potent candidates as embryo-autonomous factors of apoptosis induction. Supporting the hypothesis that the embryo autonomously induces apoptosis, a 4th-week embryo surgically extracted from the mother's body was found to initiate the apoptosis-dependent regression of the trophotaenial placenta (Supplementary Figure 2). However, this observation does not rule out the possibility of a maternal contribution. Further studies are required to determine the factors that regulate this apoptosis and whether any maternal factors are involved.

Our results also indicate that apoptotic regulation is a tissue-specific phenomenon. It could be a mechanism to prevent a hemorrhage during the regression process, which would prevent the possibility of necrotic cell death or starvation due to arrest of the circulation and nutrient transport. Another possibility is that the surviving cells contribute to vascular rearrangement during the regression process. A previous study showed that vascular networks at the perianal region of another goodeid fish, *Skiffia bilineata*, were dramatically rearranged during the perinatal stage^25^. Thus, our findings suggest that prenatal regression involves not only tissue-specific apoptotic disruption of the unnecessary processes but also serves to help remodel the vascular networks to form an alternate circulatory pathway at the perianal region. Therefore, determining the specific regulatory mechanism involved in the initiation of trophotaeniae regression is an important topic to help further understand the viviparous machinery of the goodeid fish.

## Experimental Procedures

### Animal experiments

This study was approved by the ethics review boards for animal experiments of Kyoto University. We sacrificed live animals in minimal numbers under anesthesia according to the institutional guidelines.

### Fish breeding

*Xenotoca eiseni* was purchased from Meito Suien Co., Ltd. (Nagoya-city, Japan). Adult fish were maintained in freshwater at 27°C under a 14:10-h light-dark photoperiod cycle. Fish were bred in a mass-mating design, and approximately 50 fish were maintained for this study. The juveniles were fed live brine shrimp larvae and Hikari Rabo 450 fish food (Kyorin Co., Ltd.; Himeji-city, Japan), and the adults were fed Hikari Crest Micro Pellets (Kyorin). To accurately track the pregnancy period, the laboratory-born fish were crossed in a pair-mating design, and the mating behavior was recorded.

### Embryo extraction

Pregnant females were anesthetized using tricaine on ice, following which the embryos were surgically extracted. In this study, we dissected approximately 20 pregnant females, and extracted 20–40 embryos in each operation. In order to collect the fry immediately after birth, we checked the females in late pregnancy every day. Approximately 10–30 live fry (~15 mm in length) were obtained from each delivery. Some fry were dead at birth. Microscopy observations were performed with a Leica M205C microscope (Leica Microsystems; Mannheim, Germany).

### Histology

Fish samples were fixed with Davidson's fixative solution (33% ethanol, 8% formaldehyde, and 11% acetic acid) at room temperature. Preparation of paraffin sections and HE staining were performed by following a standard protocol in our laboratory, or by Biopathology Institute Co., Ltd. (Kunisaki-city, Japan).

### Immunohistochemistry

Fish samples were fixed in 4% paraformaldehyde/phosphate-buffered saline (PBS) at 4°C overnight. Fixed samples were permeabilized using 0.5% TritonX-100/PBS at room temperature for 30 min, and then treated with Blocking-One solution (Nacalai Tesque) at room temperature for 1 h. Anti-activated-caspase3 (rabbit, Sigma-Aldrich), anti-fibronectin (rabbit, Sigma-Aldrich), and anti-εy-globin (rabbit, a gift from Dr. T. Atsumi, RIKEN; Wako-city, Japan) were used as the primary antibodies^26^. Each antibody was used in 1:500 dilution with Blocking-One solution. Samples were reacted with primary antibodies at 4°C overnight. An Alexa-488 secondary antibody (Life Technologies) was used at 1:500 dilution in 0.1% Tween-20/PBS with Alexa Fluor® 546 Phalloidin (Life Technologies) and DAPI (Sigma-Aldrich). Samples were treated in the secondary antibody solution at 4°C overnight. Microscopic observation was performed using Leica MZ16FA, Leica TCS SP5, and Leica TCS SP8 microscopes (Leica Microsystems).

### Micro-angiography

Collected fry were anesthetized on ice. A rhodamine-conjugated dextran/PBS solution was injected into the caudal vein of the fry by using a pneumatic microinjection system (NARISHIGE). Microscopy observations were performed with a Leica M205C microscope.

## Author Contributions

A.I. designed the experiments. A.I. and T.N. performed the experiments. A.I. and A.S.F. wrote the paper.

## Supplementary Material

Supplementary Information Supplementary Information

Supplementary Information Movie S1

Supplementary Information Movie S2

Supplementary Information Movie S3
