# Supplementary material for: Prenatal regression of the trophotaenial placenta in a viviparous fish, Xenotoca eiseni
Source: Sci Rep. 2015 Jan 19;5:7855. doi: 10.1038/srep07855 (PMC4297964; doi:10.1038/srep07855)
Supplement: Supplementary Information [file srep07855-s1.pdf]

Supplemental Information

Prenatal regression of the trophotaenial placenta in a viviparous fish, *Xenotoca eiseni*

Atsuo Iida, Toshiyuki Nishimaki and Atsuko Sehara-Fujisawa

The supplemental information includes supplemental experimental procedures, two supplemental figures and three supplemental movies.

Supplemental Experimental Procedures

ex vivo apoptosis induction

The 4th week embryos were surgically extracted from anesthetized pregnant female. Obtained embryo was incubated in a 60mm dish of a fresh water at 27°C until the observation. Microscope observation was performed using Leica M205C and Leica MZ16FA.

Video capturing for blood circulation

Extracted embryo was quickly anesthetized on ice, and recorded the blood circulation. Time-lapse imaging was performed using Leica MZ16FA.

Supplemental Figures

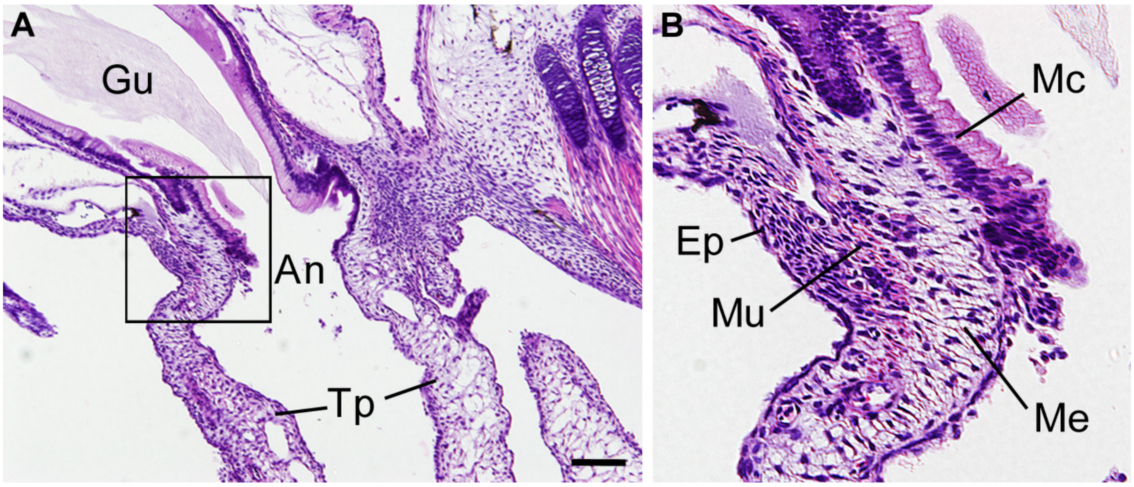

Figure S1. Histological analysis for the basal structure of the trophotaeniae.

This figure relates to Figure1. **A.** Hematoxylin-eosin-stained sagittal sectioning of the perianal region of the 3rd week embryo. The trophotaeniae were continuous with a part of the gut and the ectoderm of the embryo. An; anus, Gu; gut, Tp; trophotaenia. **B**. Enlarged image of (A). Ep; epidermis, Mc; mucosal layer, Me; mesenchyme, Mu; muscle layer.

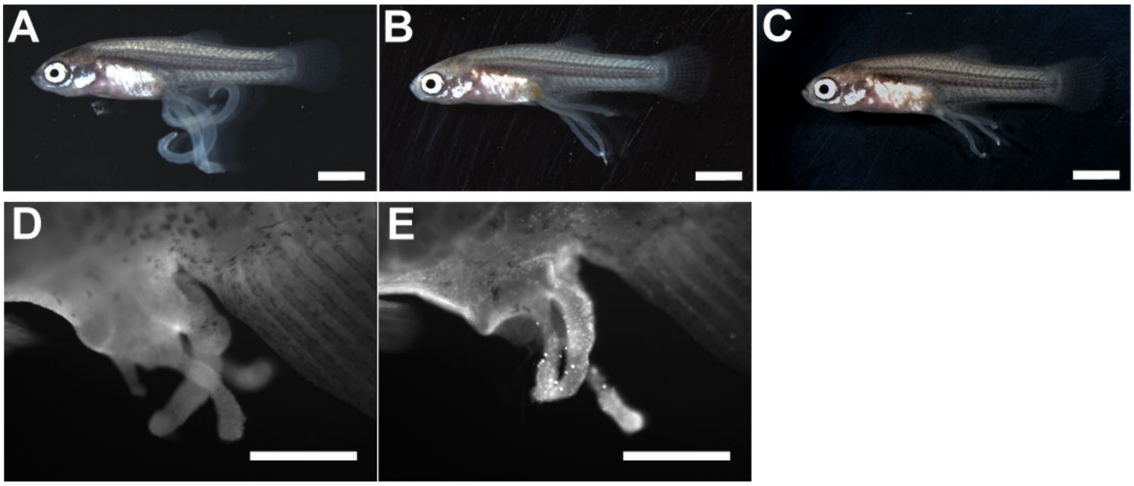

Figure S2. **Induction of the apoptotic regression in the extracted embryo.** This figure relates to Figure2. **A-C.** Time course images for *ex vivo* regression of the trophotaeniae in the 4th week embryo immediate (A), 24 hours (B), and 48 hours after the extraction. Scale bar; 2 mm. **D-E**. Fluorescent immunochemistry to detect the apoptotic cells in the *ex vivo* regressed processes. Control IgG (D) and Active-caspase-3 (E). Scale bar; 500 μm.

Supplemental Movies

Movie S1. Blood circulation of the trophotaeniae in the 3rd weeks embryo. This movie relates to Figure 1.

Movie S2. Blood circulation of the regressed processes in the fry. This movie relates to Figure 3.

Movie S3. Enlarged image of Movie S2. Turned edge of the circulation pathway in the regressed process. This movie relates to Figure3.
